# Nlrp3 Increases the Host’s Susceptibility to Tularemia

**DOI:** 10.3389/fmicb.2021.725572

**Published:** 2021-10-06

**Authors:** Ragavan V. Suresh, Elizabeth W. Bradley, Matthew Higgs, Vincenzo C. Russo, Maha Alqahtani, Wiehua Huang, Chandra Shekhar Bakshi, Meenakshi Malik

**Affiliations:** ^1^Department of Pathology, Microbiology and Immunology, New York Medical College, Valhalla, NY, United States; ^2^Department of Basic and Clinical Sciences, Albany College of Pharmacy and Health Sciences, Albany, NY, United States

**Keywords:** *Francisella tularensis*, Nlrp3, inflammasome, pro-inflammatory cytokines, virulence, IL-1β

## Abstract

*Francisella tularensis* (*F. tularensis*) is a Gram-negative, intracellular bacterium and the causative agent of a fatal human disease known as tularemia. The CDC has classified *F. tularensis* as a Tier 1 Category A select agent based on its ease of aerosolization, low infectious dose, past use as a bioweapon, and the potential to be used as a bioterror agent. *Francisella* has a unique replication cycle. Upon its uptake, *Francisella* remains in the phagosomes for a short period and then escapes into the cytosol, where the replication occurs. *Francisella* is recognized by cytosolic pattern recognition receptors, Absent In Melanoma 2 (Aim2) and *N*acht *LR*R and *P*YD domains containing Protein *3* (Nlrp3). The recognition of *Francisella* ligands by Aim2 and Nlrp3 triggers the assembly and activation of the inflammasome. The mechanism of activation of Aim2 is well established; however, how Nlrp3 inflammasome is activated in response to *F. tularensis* infection is not known. Unlike Aim2, the protective role of Nlrp3 against *Francisella* infection is not fully established. This study investigated the role of Nlrp3 and the potential mechanisms through which Nlrp3 exerts its detrimental effects on the host in response to *F. tularensis* infection. The results from *in vitro* studies demonstrate that Nlrp3 dampens NF-κB and MAPK signaling, and pro-inflammatory cytokine production, which allows replication of *F. tularensis* in infected macrophages. *In vivo*, Nlrp3 deficiency results in differential expression of several genes required to induce a protective immune response against respiratory tularemia. Nlrp3-deficient mice mount a stronger innate immune response, clear bacteria efficiently with minimal organ damage, and are more resistant to *Francisella* infection than their wild-type counterparts. Together, these results demonstrate that Nlrp3 enhances the host’s susceptibility to *F. tularensis* by modulating the protective innate immune responses. Collectively, this study advances our understanding of the detrimental role of Nlrp3 in tularemia pathogenesis.

## Introduction

*Francisella tularensis* (*F. tularensis*) is a Gram-negative, intracellular bacterium and the causative agent of a fatal human disease known as tularemia. *Francisella tularensis* strains classified under subsp. *tularensis* (Type A) and *holarctica* (Type B) are virulent, while those classified under subsp. *novicida* (*F. novicida*) and *mediasiatica* do not cause disease in immunocompetent humans (Mörner et al., 1993). Except for *F. mediasiatica*, the other three *Francisella* subspecies are highly virulent in mice and cause an acute respiratory infection upon intranasal (i.n.) challenge that recapitulates symptoms of human tularemia. *Francisella tularensis* was used in bioweapon programs of several countries in the past and is now considered a potential bioterror agent (Altman, 2002; Hirschmann, 2018). Based on its bioweapon and bioterror potential, the CDC has classified *F. tularensis* as a tier 1 category A select agent. The live vaccine strain (LVS) was developed in the former Soviet Union in the 1950s from type B *F. tularensis* subspecies *holarctica*. *Francisella tularensis* LVS is attenuated for virulence in humans, and this attenuation was achieved by multiple *in vivo* and *in vitro* passages (Eigelsbach and Downs, 1961). *Francisella tularensis* LVS shows a high degree of sequence homology, a very similar replication cycle to *F. tularensis* Type A strains and can be handled in Biosafety level 2 (BSL2) laboratories. Therefore, *F. tularensis* LVS is used as a surrogate to highly virulent type A strains in the research laboratories to study *Francisella* virulence factors and pathogenesis (Jones et al., 2014).

*Francisella* is an intracellular pathogen and can infect several cell types, including phagocytic cells such as macrophages, neutrophils, and dendritic cells (Hall et al., 2007; Craven et al., 2008a). However, infection of macrophages initiates the infection. *Francisella* has a unique intracellular life cycle. *Francisella* is taken up by the phagocytic cells by endocytosis or receptor-mediated uptake. *Francisella* stays in the phagosomes for a short period, prevents phagosomal maturation, and then escapes into the cytosol by rupturing the phagosomal wall (Santic et al., 2006). Since the replication occurs in the cytosol, the cytosolic pattern recognition receptors assume great significance in the induction of effective innate immune responses against *Francisella*.

An interferon gamma-inducible HIN-200 family member known as absent in Melanoma 2 (Aim2) activates the inflammasome by recognizing cytosolic DNA. *Francisella novicida* and *F. tularensis* are recognized primarily by the Aim2 inflammasome. Recognition of *Francisella* DNA by Aim2 results in recruitment of adaptor protein ASC and pro-caspase1 to form a multiprotein complex known as inflammasome. Activation of Aim2 inflammasome cleaves pro-caspase1 into active caspase1, which in turn cleaves pro-forms of IL-1β and pro-IL-18 into their active forms IL-1β and IL-18. Caspase1 also cleaves gasdermin D (GSDMD) into N- and C-terminal domains (Banerjee et al., 2018). The N-terminal fragment of GSDMD forms pores in the cell membrane, which allows secretion of IL-1β and IL-18, potassium efflux, and pyroptotic cell death. Studies conducted with *F. novicida* have demonstrated that ASC or caspase-1 deficient mice exhibit increased morbidity, mortality, and higher bacterial burden. Furthermore, Aim2-deficiency and the neutralization of IL-1β and IL-18 enhance susceptibility to *F. novicida* infection (Mariathasan et al., 2006; Jones et al., 2010; Belhocine and Monack, 2012). Collectively, these studies have established a significant role of Aim2 in protective innate immune responses against *Francisella*, particularly *F. novicida*.

The NLR family proteins can detect bacterial pathogen-associated molecular patterns and danger-associated molecular patterns in the cytoplasm (Harton et al., 2002; Inohara and Nuñez, 2003; Inohara et al., 2005). Upon activation, the NLR proteins NLRP1 (or NALP1), cryopyrin or NALP3 (NLRP3), or the NLRC4 (IPAF) assemble with ASC and procaspase-1 to form an inflammasome. Nlrp3 is the most studied NLR family protein. Nlrp3 is activated in response to pathogenic microorganisms, ATP, particulate matters such as asbestos, silica, uric acid crystals, potassium efflux, and lysosomal damage associated with cathepsin B release (Mariathasan et al., 2006; Martinon et al., 2006; Pétrilli et al., 2007; Cassel et al., 2008; Dostert et al., 2008, 2009; Pedra et al., 2009). All these stimuli generate reactive oxygen species (ROS), which in turn triggers Nlrp3-inflammasome activating signals such as PI3K and thioredoxin interacting protein (TXNIP; Zhou et al., 2010). Studies have shown that Nlrp3 is activated in a ligand-receptor independent but ROS-dependent manner following infection with many pathogens (Dostert et al., 2009; Joly et al., 2009; Thomas et al., 2009; Jin and Flavell, 2010; Saïd-Sadier et al., 2010). *Francisella* is also detected by Nlrp3. However, the mechanism of activation of Nlrp3 inflammasome in response to *F. tularensis* infection is not known. An initial study reported that in response to *F. novicida* infection, both NLRP3 and AIM2 inflammasomes are activated in human cells. However, only Aim2 inflammasome-dependent IL-1β production was observed in mouse macrophages (Periasamy et al., 2016). Later studies demonstrated that both Nlrp3 and Aim2 are required for IL-1β production in murine macrophages infected with *F. tularensis* LVS and the virulent type A SchuS4 strain (Duffy et al., 2016; Periasamy et al., 2016). Nlrp3 also contributes to the IgA response vital for protection against *F. tularensis* LVS challenge in immunized mice (Duffy et al., 2016). However, the activation of Aim2 and Nlrp3-inflammasomes are repressed in macrophages infected with *F. tularensis* LVS, and both are dispensable for vaccine-induced immunity against pulmonary tularemia (Atianand et al., 2011; Dotson et al., 2013; Wallet et al., 2016; Alqahtani et al., 2021). It has been reported that Nlrp3 exacerbates the symptoms of pulmonary tularemia primarily by causing excessive infiltration of immature neutrophils in the lungs of mice infected with *F. tularensis*, indicating a pathogenic role for Nlrp3 (Periasamy et al., 2016). This study investigated the role of Nlrp3 and the potential mechanisms through which Nlrp3 exerts its detrimental effects on the host in response to *F. tularensis* infection.

## Materials and Methods

### Bacterial Strains and Culture Conditions

*Francisella tularensis* Live Vaccine Strain (*F. tularensis* LVS) and *Salmonella* Typhimurium strains were obtained from BEI Resources Manassas, VA. The *Francisella* cultures were grown on Mueller-Hinton (MH) chocolate agar plates (BD Biosciences, San Jose, CA) supplemented with IsoVitaleX at 37°C with 5% CO_2_ or in MH broth (BD Biosciences, San Jose, CA) supplemented with ferric pyrophosphate and IsoVitaleX (BD Biosciences, San Jose, CA) at 37°C with shaking (160rpm). Luria-Bertani broth or McConkeys agar was used for culturing *S*. Typhimurium. All bacterial culture stocks grown to the mid-log phase were stored at −80°C and aliquots were thawed for use in experiments. All bacteria were handled in a Bio-Safety Level-2 cabinet.

### Mice and Cell Lines

Six-to-eight-week-old wild-type and *Nlrp3*^−/−^ mice of either sex on a C57BL/6 background were purchased from Jackson Laboratory (Bar Harbor, ME). All mice were maintained in a specific pathogen-free environment in the Animal Resource Facility of New York Medical College. All animal work was conducted in accordance with the protocols approved by New York Medical College’s Institutional Animal Care and Use Committee.

Immortalized bone marrow-derived macrophages (BMDMs) from wild type, *caspase1*, *Asc*, *Nlrp3*, and *Aim2* deficient mice on a C57BL/6 background were a kind gift from Dr. Katherine Fitzgerald, UMass Worcester, MA. These immortalized cell lines were generated using gene trap insertions and have been used in our previous studies (Dotson et al., 2013). Primary murine BMDMs were isolated from 6- to 8-week-old wild-type and *Nlrp3*^−/−^ mice on a C57BL/6 background purchased from Jackson Laboratory (Bar Harbor, ME) as previously described (Mahawar et al., 2012). These were differentiated into macrophages by culturing in Dulbecco’s Modified Eagle Medium (DMEM) supplemented with 10% heat-inactivated Fetal Bovine Serum (FBS), 2% l-glutamine, 1% sodium pyruvate, 1% HEPES, and 20% medium conditioned by L929 cells.

### Cell Culture Assays

Bacterial replication in infected wild type, *Asc*^−/−^, *Caspase1*^−/−^, and *Nlrp3*^−/−^ macrophages was quantified by performing gentamicin protection assays as previously described (Rabadi et al., 2016; Alqahtani et al., 2018; Alharbi et al., 2019). The macrophages were infected with *F. tularensis* LVS at a multiplicity of infection (MOI) of 100:1 (Bacteria/cell ratio). In a similar experiment, wild-type and *Nlrp3*^−/−^ macrophages were infected with *S*. Typhimurium. Cells were lysed and intracellular bacteria were enumerated at 4 and 24h post-infection. Bacterial numbers were expressed as Log_10_ CFU/ml.

For macrophage assays using MCC950, prior to treating primary BMDMs with MCC950, the metabolic state of the BMDMs was synchronized by starving the cells. To achieve this, the existing media was removed and replaced with 1ml of pre-warmed serum-free DMEM supplemented with 1μl of 10μM MCC950 dissolved in DMSO or 1μl of DMSO for vehicle control. BMDMs were incubated in this starvation media for 1h at 37°C, 5% CO_2_. One hour post-incubation, the DMEM was replaced with complete BMDM media supplemented with DMSO or MCC950, and the gentamycin protection assays were performed as described above.

### Western Blotting

Western blotting analyses to detect levels of IκBα, ERK1/2, caspase1, and IL-1β were carried out as described previously (Mahawar et al., 2012; Dotson et al., 2013; Rabadi et al., 2016). Wild type, *Aim*2^−/−^, or *Nlrp3*^−/−^ macrophages were infected with *F. tularensis* LVS at an MOI of 100 for 24h. Cells were lysed at this time and protein concentrations were normalized. Equal amounts of cell lysate were then resolved on 10% SDS-PAGE gels and transferred to polyvinylidene fluoride membranes and probed using antibodies specific to total and bio-active IκBα, ERK1/2, caspase 1, and IL-1β. The blots were developed and resulting bands quantified as previously described using Bio-rad Lab™ software (Dotson et al., 2013). Blots were then stripped and re-probed for β-actin to confirm the accuracy of protein loading.

### Mice Infection, Histopathology, and Cytokine Measurement

Wild-type and *Nlrp3*^−/−^ mice were deeply anesthetized and infected intranasally (i.n.) with 0.6×10^4^CFU (sub-lethal dose), 1×10^4^CFU, or 1×10^5^CFU of *F. tularensis* LVS. Mice were observed for morbidity by weighing periodically and mortality for 21days. The results were expressed as Kaplan-Meier survival curves and statistical significance was established by the Log-rank test. To study bacterial clearance, wild-type and *Nlrp3*^−/−^ mice were infected i.n. with 1×10^4^CFU of *F. tularensis* LVS and killed on days three and seven post-infection. For studying *in vivo* role of MCC950, wild-type mice were injected IP with 200μl of MCC950 at a concentration of 10mg/kg body weight for 7days and were then challenged with 1.5×10^4^ CFUs of *F. tularensis* LVS intranasally. Lungs, livers, and spleens were isolated aseptically and homogenized using sterile zirconia beads, serially diluted, and plated onto MH chocolate agar plates to quantify bacterial burdens in these organs. Small portions of the organs were also fixed in 10% formalin, embedded in paraffin, sectioned, and stained with hematoxylin and eosin for histopathological evaluation and scoring as previously described (Bakshi et al., 2008). The resulting colonies were counted and bacterial burden was represented as Log_10_ CFU/ml. Lung homogenates collected from wild-type and *Nlrp3*^−/−^ mice on days three and seven post-infection with 1×10^4^CFU of *F. tularensis* LVS were clarified by centrifugation and assayed for a panel of 23 cytokines using “Bio-plex Pro mouse cytokine 23-plex assay” (Biorad). Cytokine levels were expressed as picograms/ml (pg/ml).

### Immunofluorescence Microscopy

The subcellular localization of p65 subunit of NF-κB was determined by immunofluorescence microscopy as previously described (Mahawar et al., 2012; Rabadi et al., 2016). Wild-type and *Nlrp3*^−/−^ macrophages were infected for 2h and processed for staining. At least 100 cells were counted at random to quantify the nuclear or cytosolic localization of p65.

### RNA Extraction and Sequencing

Wild-type and *Nlrp3^−/−^* mice infected with 1×10^4^ *F. tularensis* LVS were sacrificed on days 3 and 7 post-infection. Lungs, livers, and spleens were harvested aseptically from these mice and placed in excess of trizol. Organs were snap-frozen in liquid nitrogen and stored at −80°C until required. When ready, the organs frozen in trizol were thawed on ice and homogenized in 2ml of fresh trizol using a tissue-tearor. The homogenate was centrifuged at 15,000×*g* for 15min at 4°C to pellet the tissue debris and limit RNA degradation from tissue-resident nucleases. Total RNA was extracted from the clarified supernatants using the “Purelink RNA mini kit” (Invitrogen) following the manufacturer’s instructions. DNA was eliminated from the samples by DNAse treatment using “RNAse-Free DNAse” (Qiagen). Purified RNA was eluted in water and stored at −80°C.

Prior to RNA sequencing, total RNA extracted from mice had to be assayed for quantity and quality. RNA was quantified using Qubit RNA HS Assay kit on a Qubit fluorometer (Thermo Fisher). The quality of the RNA samples was measured in terms of RNA Integrity Number (RIN), which is a measure of sample degradation and strand breakage. RIN for samples was calculated using the 4200 Tapestation (Agilent) and samples with RIN>7 were considered intact and used for further RNA sequencing. Sequencing libraries were generated using TruSeq Stranded mRNA kit and murine RNA sequencing was performed on a Next Seq 550 (Illumina) and data were represented as transcripts per million (TPM). Transcript levels from wild-type mice were and *Nlrp3^−/−^* mice were quantified and compared on days 3 and 7 post-infection using Salmon software (Patro et al., 2017). Salmon was also used to compare the change in transcript levels within the groups from day 3 to day 7 post-infection. Significance was established using the Wald log test and differences were considered significant at a *q* value <0.05. Functional annotation and clustering of genes were performed using DAVID Bioinformatics Resources 6.8 (Huang et al., 2009a,b).

### Statistical Analysis

All results were expressed as means±SD or mean±SEM. Statistical comparisons between the groups were made using unpaired *t*-tests or ANOVA with the Tukey-Kramer test. Survival results were plotted as Kaplan-Meier survival curves, and significance was calculated using the Log-rank test. Differences between the experimental groups were considered statistically significant at a *p*<0.05 level.

## Results

### Nlrp3 Deficiency Results in Higher Levels of Pro-inflammatory Cytokines

**Figure 1:**
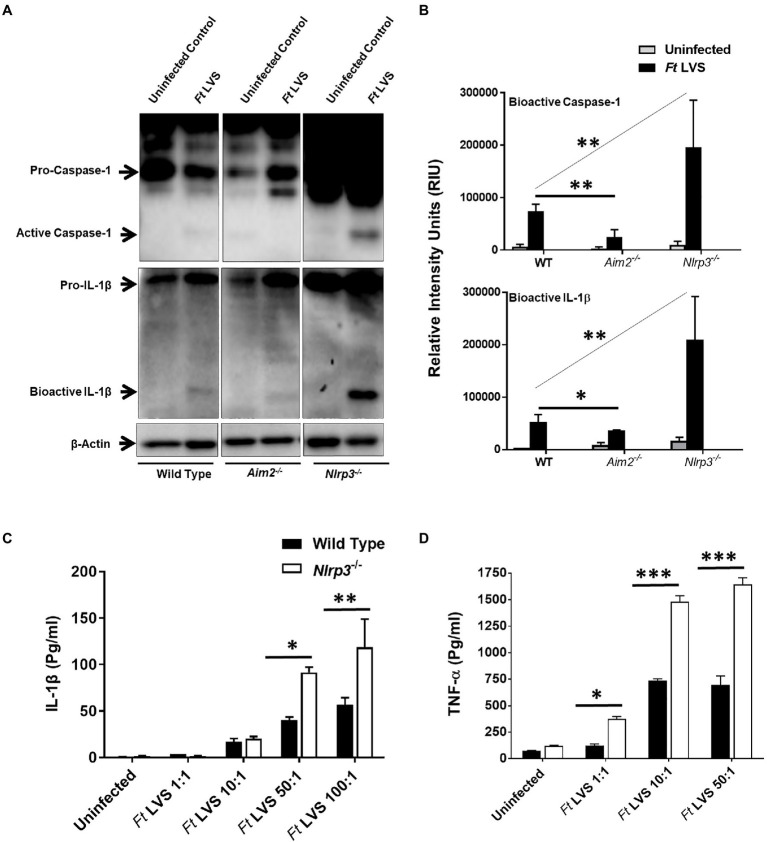
Nlrp3 dampens pro-inflammatory cytokine response. **(A)** Wild type, *Aim2^−/−^* and *Nlrp3^−/−^* macrophages were infected with *Francisella tularensis* (*Ft*) live vaccine strain (LVS) at an multiplicity of infection (MOI) of 100. The cell lysates were prepared 24h post-infection and western blot analysis was performed to determine levels of bioactive caspase1 and IL-1β. The western blot images are representative of three independent experiments. β-actin was used as a loading control. The uncropped version of **(A)** is shown in Supplementary Figure S1. **(B)** The quantification of the bands (*n*=3 blots) is shown and expressed as relative intensity units (RIU). **(C,D)** The wild-type and the *Nlrp3^−/−^* macrophages were infected with the indicated MOI of *Ft* LVS and treated with gentamicin to kill extracellular and adherent bacteria after 2h of infection and the infection with intracellular bacteria was allowed to proceed for 22h. The IL-1β **(C)** and TNF-α **(D)** levels were measured in the culture supernatants after 24h post-infection. The results are representative of three independent experiments. The data in **B–D** were analyzed by ANOVA with the Tukey-Kramer test, and a *p*-value of 0.05 or less was considered significant. ^*^p<0.05; ^**^p<0.01; ^***^p<0.001.

Activation of Aim2 and Nlrp3 inflammasomes results in the activation of caspase1 and cleavage of pro-IL-1β into its secreted mature form IL-1β. The cleaved bioactive forms of both caspase1 and IL-1β can be detected by western blotting. We first investigated the state of inflammasome activation in the wild type, *Aim2*^−/−^ and *Nlrp3*^−/−^ macrophages infected with *F. tularensis* LVS. Wild type, *Aim2*^−/−^ and *Nlrp3*^−/−^ macrophages were infected with 100 MOI of *F. tularensis* LVS (1×10^7^ CFUs of bacteria:1×10^5^ macrophages in a 12 well-plate). The infection was allowed to progress for 2h, and then the cells were treated with gentamicin for 1h to kill all extracellular and adherent bacteria. After 24h post-infection, the cell lysates were analyzed for bioactive levels of capsase1 and IL-1β by western blot analysis. In *F. tularensis* LVS-infected wild-type macrophages, consistent with previous reports (Dotson et al., 2013), we observed very low levels of bioactive caspase1 and IL-1β. The activation of both caspase1 and IL-1β was reduced significantly in the *Aim2*^−/−^ compared to the wild-type macrophages infected with *F. tularensis* LVS. However, significantly higher levels of bioactive caspase1 and IL-1β were observed in the *Nlrp3*^−/−^ compared to the wild-type and *Aim2*^−/−^ macrophages (Figures 1A,B and Supplementary Figure S1). We next investigated the levels of secreted IL-1β in the culture supernatants from the wild-type and *Nlrp3*^−/−^ macrophages infected with a varying multiplicity of *F. tularensis* LVS and treated with gentamicin to kill all the extracellular bacteria. The cell culture supernatants were collected 24h post-infection to determine the levels of IL-1β and TNF-α exclusively in response to the intracellular bacteria. A dose-dependent increase in the levels of both IL-1β and TNF-α were observed in the *Nlrp3*^−/−^ macrophages. These levels were significantly higher than those observed in culture supernatants from the wild-type macrophages (Figures 1C,D). These results indicate that activation of caspase1 and IL-1β in macrophages infected with *F. tularensis* LVS is partially dependent on Aim2. However, Nlrp3 is dispensable. Its loss, on the contrary, is associated with increased levels of bioactive forms of both the Caspase1 and IL-1β, and pro-inflammatory cytokines IL-1β and TNF-α in macrophages infected with *F. tularensis* LVS.

### Nlrp3 Deficiency Results in Enhanced Activation of NF-κB and MAPK Signaling

**Figure 2:**
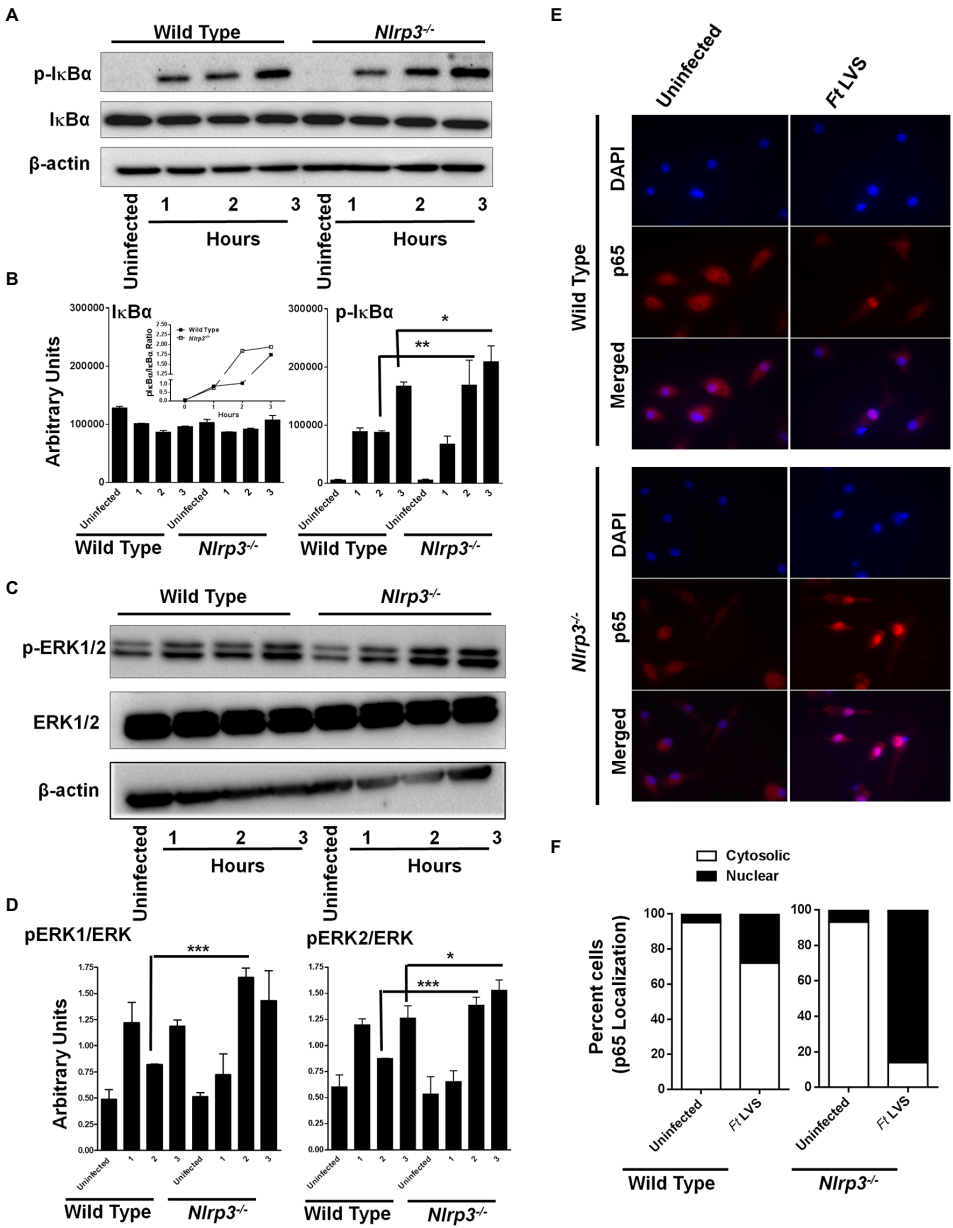
Loss of Nlrp3 results in enhanced activation of NF-κβ and MAPK signaling. The wild-type and the *Nlrp3^−/−^* macrophages were infected with 100 MOI of *F. tularensis* (*Ft*) LVS for the indicated times, lysed and separated by SDS-PAGE, and immunoblotted with phosphorylated (p) and total IκBα- **(A)** and ERK1/2 **(C)** antibodies. β-actin was used as a loading control. Quantitation of IκBα, p-IκBα **(B)**, ERK, and p-ERK bands **(D)** (*n*=2 blots). Uninfected macrophages were used as controls. The inset in B shows pIκBα/IκBα ratios. The data were analyzed by one-way ANOVA, and a *p*-value of 0.05 or less was considered significant. ^*^p<0.05; ^**^p<0.01; ^***^p<0.001. **(E)** Immunofluorescence staining was performed to detect cellular localization of the p65 subunit of NF-κβ in the wild type or the *Nlrp3^−/−^* macrophages infected with 100 MOI of *Ft* LVS (magnification 63×, red, p65; blue, nucleus). **(F)** Quantification of subcellular localization of p65 subunit of NF-κβ. At least 100 cells were counted manually in randomly selected fields. The results are expressed as percent macrophages showing cytosolic or the nuclear p65 localization. The data shown are representative of two independent experiments, each conducted with three biological replicates.

NF-κB and MAPK play important roles in the production of pro-inflammatory cytokines, especially TNF-α and pro-IL-1β. We next investigated if the elevated levels of both the IL-1β and TNF-α observed in the *Nlrp3*^−/−^ macrophages were due to enhanced activation of NF-κB and MAPK signaling. The wild-type and the *Nlrp3*^−/−^ macrophages were infected with 100 MOI of *F. tularensis* LVS and treated with gentamycin to determine the response to intracellular bacteria. The cell lysates were analyzed by western blotting for the total and phosphorylated levels of IκBα and ERK1/2 after 1, 2, and 3h of infection as a measure of NF-κB and MAPK activation. No differences in the phosphorylated-IκB (p-IκB) levels were observed between the wild-type and the *Nlrp3*^−/−^ macrophages after 1h of infection with *F. tularensis* LVS. However, the levels of p-IκB were significantly higher in the *Nlrp3*^−/−^ than those in the wild-type macrophages 2 and 3h post-infection. The ratio of p-IκBα to total IκBα was also higher in *F. tularensis* LVS-infected *Nlrp3*^−/−^ macrophages (Figures 2A,B). A similar trend was also observed for pERK1/2, with significantly elevated levels being observed at 2 and 3h post-infection in the *Nlrp3*^−/−^ compared to the *F. tularensis* LVS-infected wild-type macrophages (Figures 2C,D). We further confirmed the activation of NF-κB signaling by immunofluorescence microscopy to determine the translocation of p65 subunit of NF-κB to the nucleus in the wild-type and *Nlrp3*^−/−^ macrophages infected with *F. tularensis* LVS. Ninety-five percent of uninfected wild type or the *Nlrp3*^−/−^ macrophages revealed cytosolic localization of p65 subunit of NF-κB. About 35% of wild-type macrophages infected with the *F. tularensis* LVS had p65 localized to their nuclei 2h post-infection. Contrarily, in 90% of the *Nlrp3*^−/−^ macrophages infected with *F. tularensis* LVS, p65 was found to be localized to the cell nuclei (Figures 2E,F). Collectively, these results demonstrate that loss of Nlrp3 results in enhanced activation of NF-κβ and MAPK, which might, in turn, result in elevated levels of pro-inflammatory cytokines TNF-α and IL-1β.

### *F. tularensis* LVS Fails to Replicate in *Nlrp3*^−/−^ Macrophages

**Figure 3:**
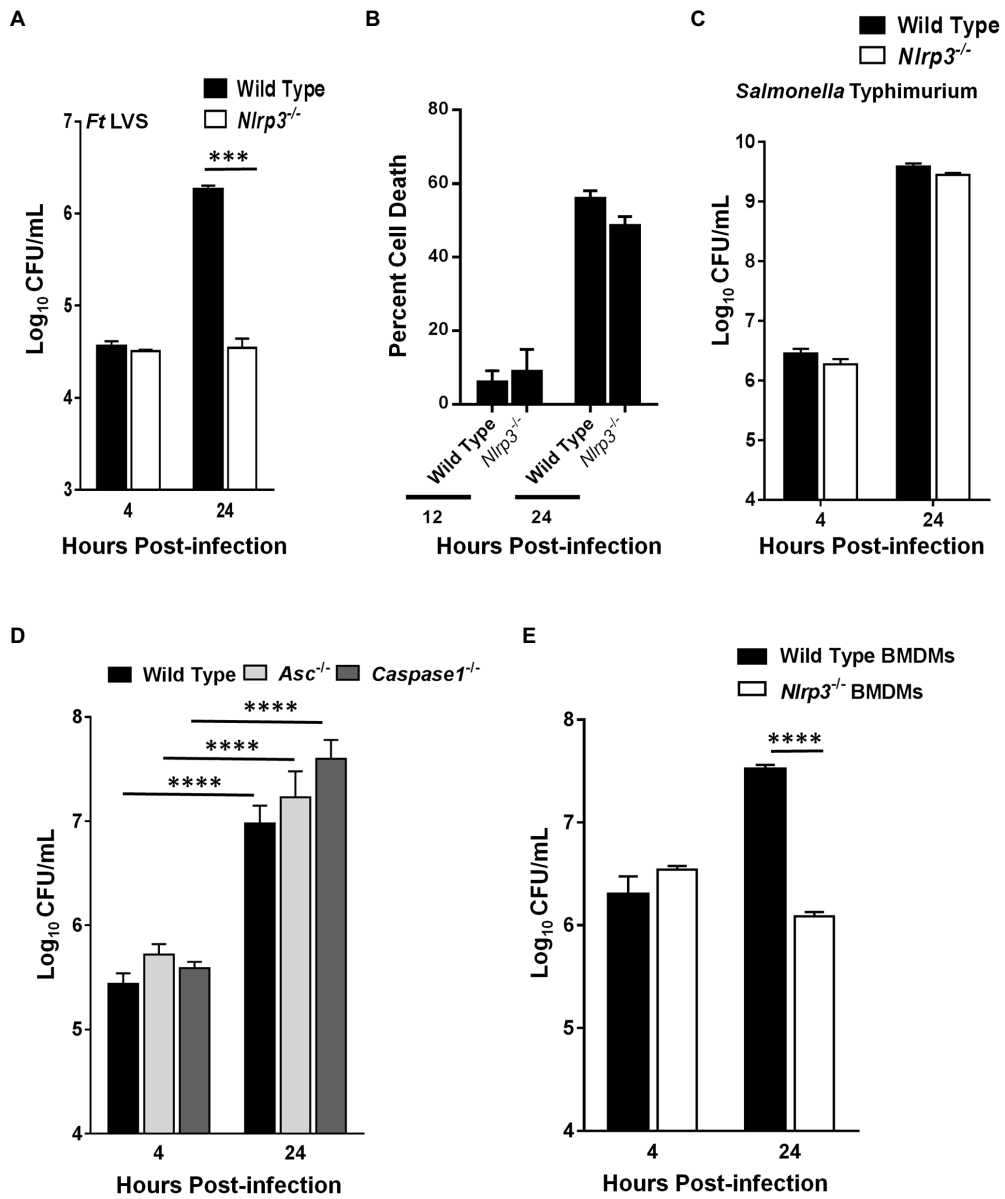
*Francisella tularensis* (*Ft*) LVS fails to replicate in *Nlrp3^−/−^* macrophages. Wild-type and *Nlrp3^−/−^* macrophages **(A,B)** were infected with 100 MOI of *Ft* LVS or **(C)** with 100 MOI of *Salmonella* Typhimurium. **(A,C)** The macrophages were lysed after 4 and 24h of infection, diluted 10-fold and plated on MH-chocolate agar plates for the enumeration of bacterial numbers. **(B)** Percent cell death was determined by LDH release assay 12 and 24h post-infection. **(D)** Wild type, *Asc^−/−^* or the *Caspase1^−/−^* macrophages were infected with 100 MOI of *Ft* LVS and bacteria were quantitated at 4 and 24h post-infection. **(E)** Wild-type and *Nlrp3^−/−^* primary bone marrow-derived macrophages (BMDMs) isolated from C57BL/6 mice were infected with 10 MOI of *Ft* LVS and bacteria were quantitated at 4 and 24h post-infection. The data are representative of two–three independent experiments each conducted with three biological replicates. The data were analyzed by ANOVA with the Tukey-Kramer test, and a value of *p* of 0.05 or less was considered significant. ^***^p<0.001; ^****^p<0.0001.

We next investigated if the elevated levels of pro-inflammatory cytokines TNF-α and IL-1β in the *Nlrp3*^−/−^ macrophages infected with *F. tularensis* also result in an enhanced bacterial clearance. The ability of *F. tularensis* to survive and replicate in *Nlrp3*^−/−^ macrophages was determined by gentamicin protection assay. Wild-type and *Nlrp3*^−/−^ macrophages were infected with 100 MOI (bacteria:cell ratio) of *F. tularensis* LVS, and intracellular bacterial numbers were quantitated at 4- and 24-h post-infection. Nearly equal numbers of bacteria were recovered from wild-type and *Nlrp3*^−/−^ macrophages at 4h post-infection, indicating similar numbers of bacteria entered the cells. After 24h, the bacterial numbers in the *Nlrp3*^−/−^ macrophages did not increase from those observed at 4h post-infection. However, in the wild-type macrophages, the bacteria replicated and nearly 100-fold more bacteria were recovered than those from the *Nlrp3*^−/−^ macrophages (Figure 3A). We next performed LDH release assays to confirm that failure of *F. tularensis* LVS to replicate in the *Nlrp3*^−/−^ macrophages was not on account of enhanced cell death of the *Nlrp3*^−/−^ macrophages following infection. The extent of cell death did not differ between the wild-type and the *Nlrp3*^−/−^ macrophages infected with *F. tularensis* LVS when observed at 12 and 24h post-infection (Figure 3B). Wild-type and *Nlrp3*^−/−^ macrophages were infected with 100 MOI of Gram-negative intracellular pathogen, *Salmonella* Typhimurium, to confirm that the *Nlrp3*^−/−^ macrophages are not defective and are capable of supporting bacterial replication. *S*. Typhimurium replicated to near equal levels in both the wild-type and the *Nlrp3*^−/−^ macrophages (Figure 3C), indicating *Nlrp3*^−/−^ macrophages are capable of supporting bacterial replication. A similar assay was performed next using the wild type, *Asc*^−/−^, and *Caspase1*^−/−^ macrophages to determine if these inflammasome components like Nlrp3 also impair the clearance of *F. tularensis* LVS. The numbers of bacteria recovered from *Asc*^−/−^ and *Caspase1*^−/−^ macrophages were significantly higher than those from the wild-type macrophages, indicating that, unlike Nlrp3, deficiency of both Asc and Caspase1 facilitate bacterial replication (Figure 3D). Like the macrophage cell lines (Figure 3A), *F. tularensis* LVS also failed to replicate in the primary BMDMs isolated from *Nlrp3*^−/−^ mice as compared to their wild-type counterparts (Figure 3E). Collectively, these results demonstrate that Nlrp3 specifically impairs the clearance of *F. tularensis* LVS, and Nlrp3-deficiency is associated with an enhanced bacterial clearance from the infected macrophages.

### *Nlrp3^−/−^* Mice Infected With *F. tularensis* LVS Exhibit an Enhanced Production of Cytokines and Chemokines as Compared to the Wild-Type Mice

**Figure 4:**
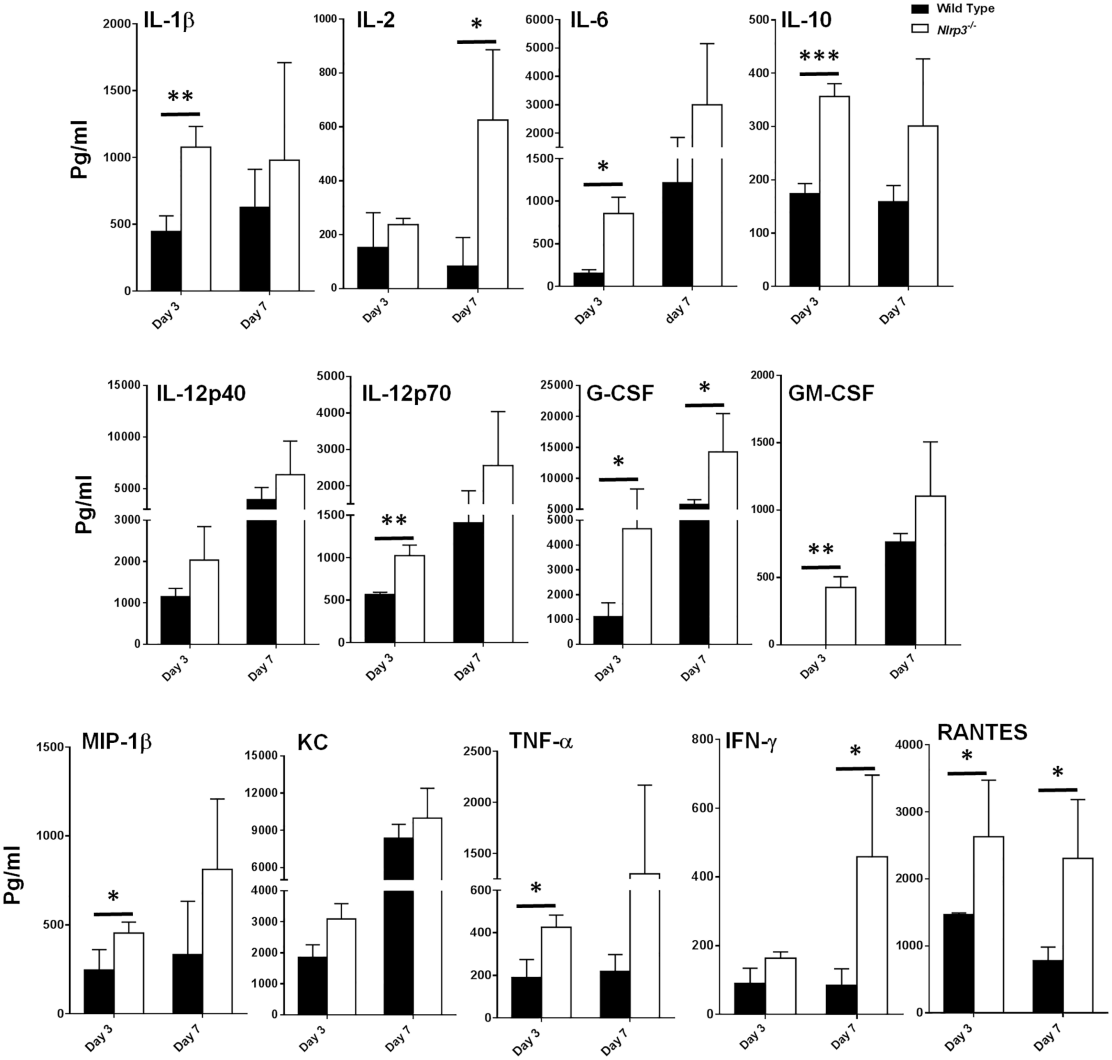
*Nlrp3^−/−^* mice exhibit enhanced production of cytokines and chemokines as compared to the wild type mice. C57BL/6 wild-type and *Nlrp3^−/−^* mice infected intranasally with 1×10^4^ CFUs of *F. tularensis* LVS were euthanized on days 3 and 7 post-infection and their lungs were homogenized. The clarified homogenates were used for quantitation of cytokines and chemokines using multiplex cytokine bead array. The cytokine levels are expressed as picogram/ml and are cumulative of two independent experiments, each conducted with *n*=3 mice/group/time point (Total *n*=6/group/time point). The data were analyzed by unpaired *t*-test and a value of *p* of 0.05 or less was considered significant. ^*^p<0.05; ^**^p<0.01; ^***^p<0.001.

Our preceding *in vitro* assays demonstrated that Nlrp3-deficiency results in elevated levels of pro-inflammatory cytokines and enhanced bacterial clearance. We corroborated our *in vitro* findings by performing *in vivo* experiments in wild-type and *Nlrp3*^−/−^ mice. C57BL/6 wild-type and *Nlrp3*^−/−^ mice were infected intranasally with 1×10^4^CFU of *F. tularensis* LVS (1×LD_100_). The cytokine levels were determined in the lung homogenates on days 3 and 7 post-infection. The lung homogenates from *Nlrp3*^−/−^ mice showed significantly higher levels of IL-1β, IL-6, IL-10, IL-12p70, G-CSF, GM-CSF, TNF-α, and RANTES than the wild-type mice on day 3 post-infection. The levels of IL-2, G-CSF, IFN-γ, and RANTES were significantly higher in the *Nlrp3*^−/−^ mice than the wild-type mice on day 7 post-infection (Figure 4). Levels of IL-1α, IL-4, IL-17, MCP-1, MIP-1α, or Eotaxin did not differ between wild-type and *Nlrp3*^−/−^ mice, while IL-3 and IL-5 remained below detection limits (data not shown). These results demonstrate that *Nlrp3*^−/−^ mice induce a potent cytokine response compared to the wild-type mice following *F. tularensis* infection, corroborating the results observed with *Nlrp3*^−/−^ macrophages.

### *Nlrp3*^−/−^ Mice Infected With *F. tularensis* LVS Harbor Fewer Bacteria and Exhibit Less Severe Histopathological Lesions Than the Wild-Type Mice

**Figure 5:**
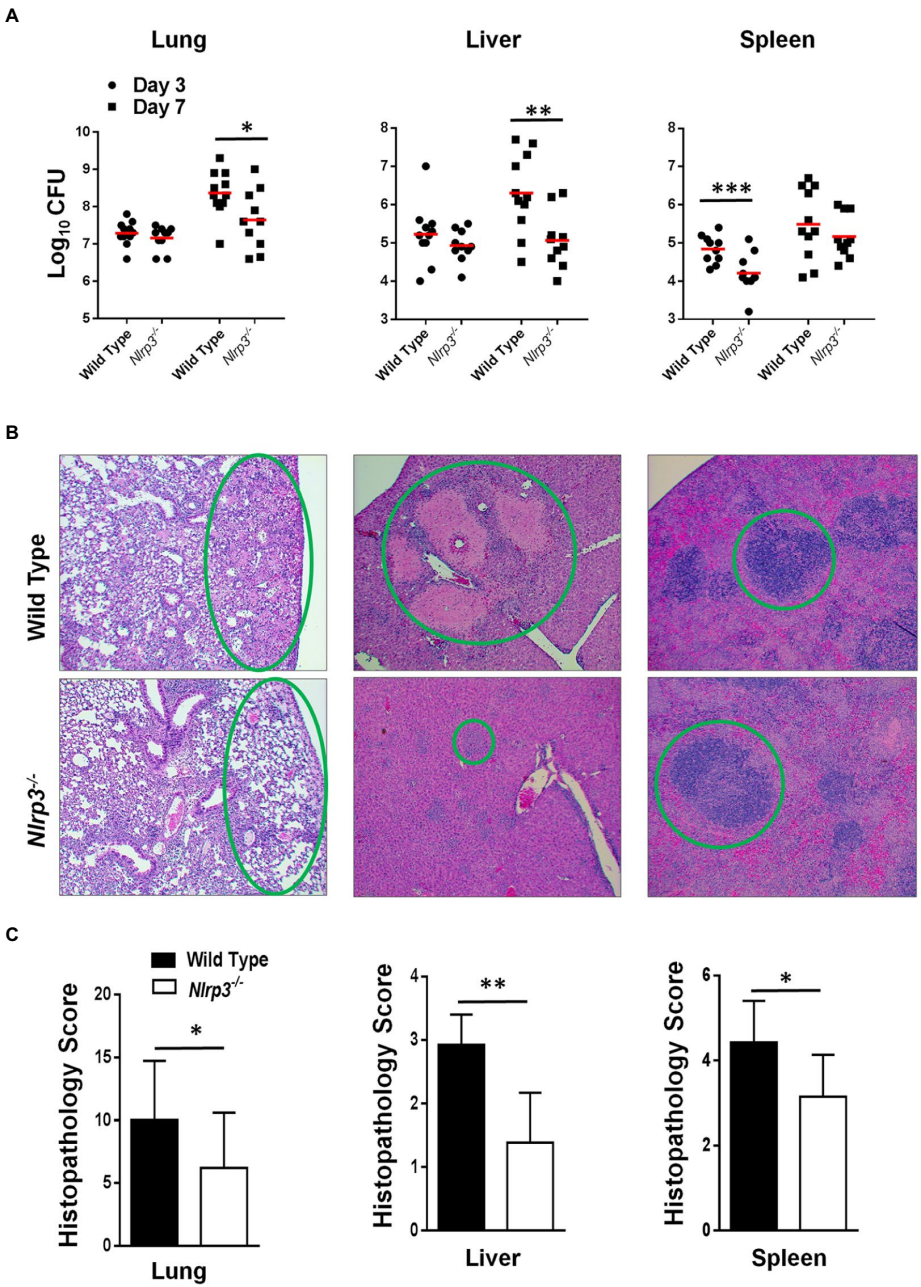
Nlrp3 deficiency results in enhanced bacterial clearance and less severe histopathological lesions in mice infected with *F. tularensis* LVS. C57BL/6 wild-type and *Nlrp3^−/−^* mice-infected intranasally with 1×10^4^ CFUs of *F. tularensis* LVS. **(A)** The mice were euthanized on days 3 and 7 post-infection and bacterial burdens were quantified in their lung, liver, and spleen. The data shown are cumulative results from three independent experiments (*n*=3–4 mice each/experiment, 11 total/time point). The data were analyzed by ANOVA with the Tukey-Kramer test, and a value of *p* of 0.05 or less was considered significant. ^**^p<0.01; ^***^p<0.001. **(B)** The lung, liver, and spleens were fixed in 10% formalin, paraffin-embedded, sliced into 5μM sections, and stained with Hematoxyline and Eosin. Stained sections from mice infected with *F. tularensis* LVS on day 7 post-infection were observed for histopathological lesions under a light microscope (Magnification 100×). The green circles compare the extent of the lesions between wild-type and *Nlrp3^−/−^* mice. **(C)** The histopathological damage in the lung, liver, and spleen was quantitated in a blinded fashion by scoring one field of view at a time. Data shown are cumulative scores from three mice and were analyzed by unpaired *t*-test. ^*^p>0.05; ^**^p>0.01; ^***^p>0.001.

The results thus far demonstrated that *Nlrp3*^−/−^ macrophages clear bacteria more efficiently than their wild-type counterparts and that the levels of pro-inflammatory cytokines are higher in both *Nlrp3^−/−^* macrophages and mice following infection with *F. tularensis* LVS. Wild-type and *Nlrp3^−/−^* mice were infected intranasally with 1×10^4^CFU of *F. tularensis* LVS. The infected mice were sacrificed on days 3 and 7 post-infection to determine the kinetics of bacterial clearance and evaluation of the histopathological lesions in the lungs, liver, and spleen. On day 3 post-infection, wild-type and *Nlrp3^−/−^* mice had similar bacterial burdens in their lungs, livers, while the *Nlrp3^−/−^* mice harbored significantly fewer bacteria in their spleens (Figure 5A). On day 7 post-infection, significantly fewer bacteria were recovered from the lungs and livers of the *Nlrp3^−/−^* compared to the wild-type mice. These results corroborated findings from *in vitro* studies where *Nlrp3*^−/−^ macrophages cleared *F. tularensis* LVS better than their wild-type counterparts.

**Figure 6:**
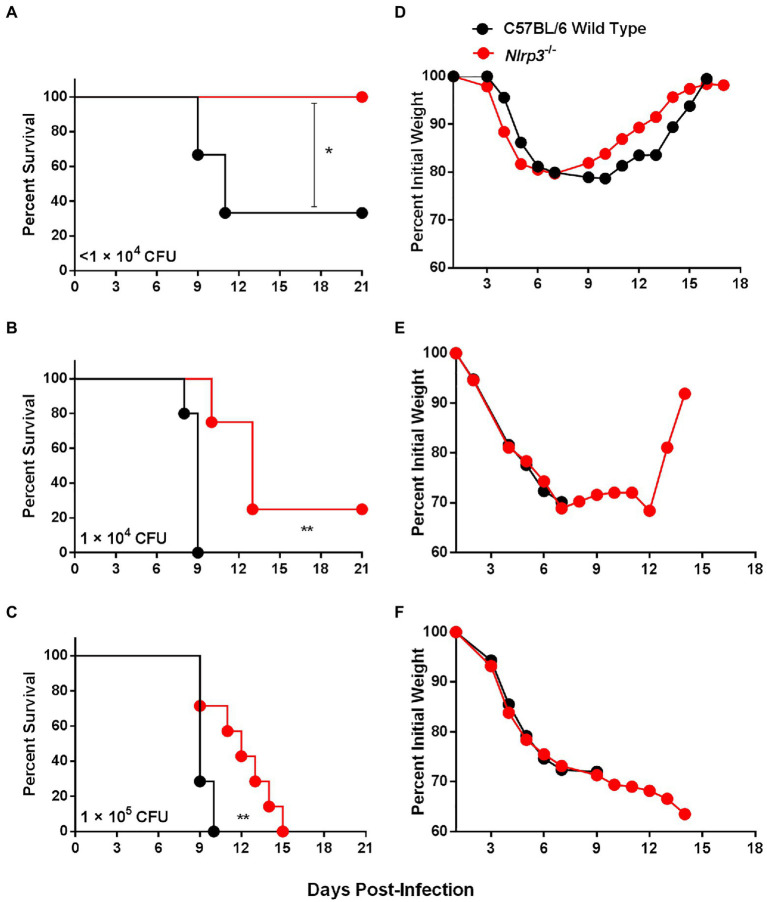
Nlrp3 deficiency results in enhanced survival of *F. tularensis* LVS-infected mice. **(A–C)** Survival graph of C57BL/6 wild-type and *Nlrp3^−/−^* mice (*n*=6–8 mice/group) infected intranasally with indicated doses of *F. tularensis* LVS. The mice were observed for mortality for 21days. The data were analyzed by the Log-rank test and values of *p* were calculated. **(D–F)** The infected mice were weighed at the indicated times to monitor morbidity. ^*^p<0.05; ^**^p<0.01.

We next quantified histopathological lesions in these mice to determine the severity of tissue damage. Sections of the organs harvested from wild-type and *Nlrp3^−/−^* mice infected with 1×10^4^CFU of *F. tularensis* LVS were stained with H&E and scored for histopathological lesions at 100× magnification in a blinded fashion. We observed that wild-type mice had significantly more inflammatory infiltrates, necrotic lesions, and consolidation of airspaces. In contrast, *Nlrp3^−/−^* mice exhibited intact alveolar spaces (Figure 5B). In the livers of wild-type mice, numerous large necrotic granulomas were observed. On the contrary, the livers of *Nlrp3^−/−^* mice displayed much smaller granulomas, evidenced by significantly lower histopathological scores (Figures 6B,C). In the spleen of wild-type mice, mild disruption of germinal centers and peri-lymphoid infiltration of red pulp was observed. The spleens of *Nlrp3^−/−^* mice exhibited similar red pulp infiltration, but the germinal centers remained largely conserved (Figure 5B). Overall, the lesions in the lung, liver, and spleens of the *Nlrp3^−/−^* mice were significantly less severe than wild-type mice (Figure 5C). These results suggest that *Nlrp3^−/−^* mice clear bacteria more efficiently than the wild-type controls and exhibit less severe histopathological lesions in the lungs, liver, and spleen.

### *Nlrp3^−/−^* Mice Are Better Protected Than the Wild-Type Mice Against Intranasal Challenge With *F. tularensis* LVS

We corroborated our *in vitro* findings by performing *in vivo* experiments in C57BL/6 wild-type and *Nlrp3^−/−^* mice. Mice were infected i.n. with 0.6×10^4^, 1×10^4^, and 1×10^5^ CFUs of *F. tularensis* and monitored for morbidity by measuring the body weights and for mortality by observing survival for 21days. One hundred percent of *Nlrp3^−/−^* mice survived the infection dose of 0.6×10^4^ CFUs, while only 33% (2/6 mice) wild-type mice survived this infection dose (Figure 6A). At a higher infection dose of 1×10^4^ CFUs, all wild-type mice succumbed to infection by day 9 post-infection; however, 25% (2/8) of the *Nlrp3^−/−^* mice survived this infection dose with an enhanced median survival time (MST) of 13days compared to 9days for the wild-type mice (Figure 6B). One hundred percent of both the wild-type and *Nlrp3^−/−^* mice infected with 1×10^5^ CFUs of the *F. tularensis* LVS succumbed to infection. However, *Nlrp3^−/−^* mice exhibited a significantly extended MST of 12days compared to 9days for the wild-type mice (Figure 6C). The infected *Nlrp3^−/−^* mice lost body weight for up to 6days post-infection, and those that survived regained their initial body weight. A similar trend was also observed for the wild-type mice that survived the infection (Figures 6D–F). Collectively, these results demonstrate that at all infection doses of *F. tularensis* LVS tested, the *Nlrp3*^−/−^ mice survived better than the wild-type mice, further cementing the notion that Nlrp3 enhances the susceptibility to *F. tularensis* infection.

### The Detrimental Role of Nlrp3 Is Independent of Its Role as an Inflammasome Activator

**Figure 7:**
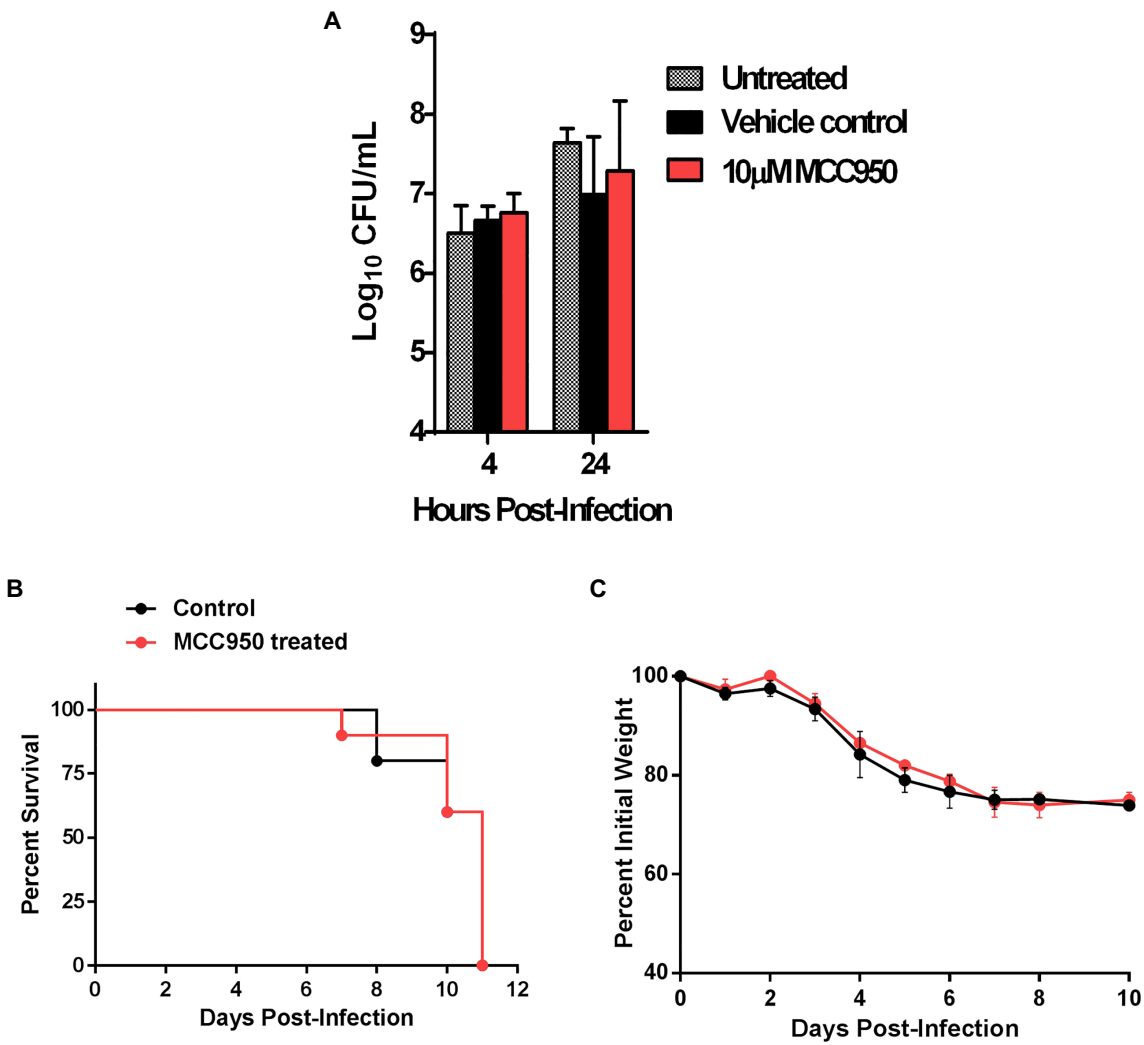
The detrimental role of Nlrp3 is independent of its role as an inflammasome activator. **(A)** Primary BMDMs isolated from C57BL/6 mice were either left untreated or treated with 10μM of MCC950 or an equal volume of DMSO as vehicle control. The BMDMs were infected with *F. tularensis* LVS at an MOI of 10 and bacterial numbers were quantitated 4 and 24h post-infection. The results are expressed as Log_10_ CFU/ml and are cumulative of two independent experiments each conducted with 2–3 biological replicates (*n*=5 total). C57BL/6 mice (*n*=10) were injected intraperitoneally (i.p.) with MCC950 (10mg/kg body weight) for six consecutive days. The control mice (*n*=10) were injected i.p. with an equal volume of DMSO as vehicle controls. All mice were infected i.n. with 1.5×10^4^CFU of *F. tularensis* LVS and were observed for survival and weight loss. Survival results are plotted as Kaplan-Meir survival curves **(B)** and the body weights are represented as percent initial body weight **(C)**.

We next investigated if the detrimental effect of Nlrp3 in response to primary infection with *F. tularensis* is dependent on its role as an inflammasome activator. We used a small molecule inhibitor, MCC950, to selectively disrupt the Nlrp3 inflammasome without affecting its inflammasome-independent functions (Coll et al., 2019). Primary BMDMs isolated from C57BL/6 wild-type mice were seeded in DMEM containing 10μM MCC950 or an equal volume of DMSO as controls. The BMDMs were infected with 10 MOI of *F. tularensis* LVS and were lysed at 4 and 24h post-infection to count intracellular bacteria. No significant differences were observed in bacterial numbers recovered at 24h post-infection from MCC950 treated (7.28±0.88 Log_10_ CFU/ml), vehicle control (6.94±0.73 Log_10_ CFU/ml), or untreated (7.64±0.17 Log_10_ CFU/ml) macrophages (Figure 7A). These results show that, unlike Nlrp3-deficient macrophages, the inhibition of Nlrp3 inflammasome in the wild-type macrophages does not affect bacterial clearance.

We next investigated if similar results could be replicated *in vivo*. Six-to-eight-week-old C57BL/6 mice were injected intraperitoneally with 10mg/kg of MCC950 for six consecutive days. These mice were then infected i.n. with 1.5×10^4^CFU of *F. tularensis* LVS on day 2 of the treatment. Mice injected with DMSO were used as vehicle controls and observed for morbidity and mortality. Both the MCC950 treated and control mice succumbed to the infection by day 11 post-infection. There were no differences in body weight loss patterns between the two groups of mice (Figures 7B,C). Collectively, these results demonstrate that the detrimental effects of Nlrp3 in response to *F. tularensis* LVS infection are independent of inflammasome-related functions.

### Genes Known to Enhance the Host’s Susceptibility to *F. tularensis* Infection Are Downregulated in *Nlrp3^−/−^* Mice

Results from our preceding studies demonstrate a detrimental inflammasome-independent role of Nlrp3 in response to *F. tularensis* infection. Recent reports have suggested that in addition to the inflammasome-dependent function, Nlrp3 also serves as a transcriptional regulator (Bruchard et al., 2015; Wang et al., 2016a). We next investigated if the detrimental effects of Nlrp3 are due to its impact on the expression of various genes in mice infected with *F. tularensis* LVS. RNA-sequencing was performed to determine the transcript levels of genes using total RNA extracted from lungs of wild-type C57BL/6 and *Nlrp3^−/−^* mice infected with 1×10^4^CFU of *F. tularensis* LVS on days 3 and 7 post-infection. Pairwise analysis of transcripts from C57BL/6 wild-type and *Nlrp3^−/−^* mice on days 3 and 7 post-infection was performed to determine differentially expressed genes between these strains of mice.

**Figure 8:**
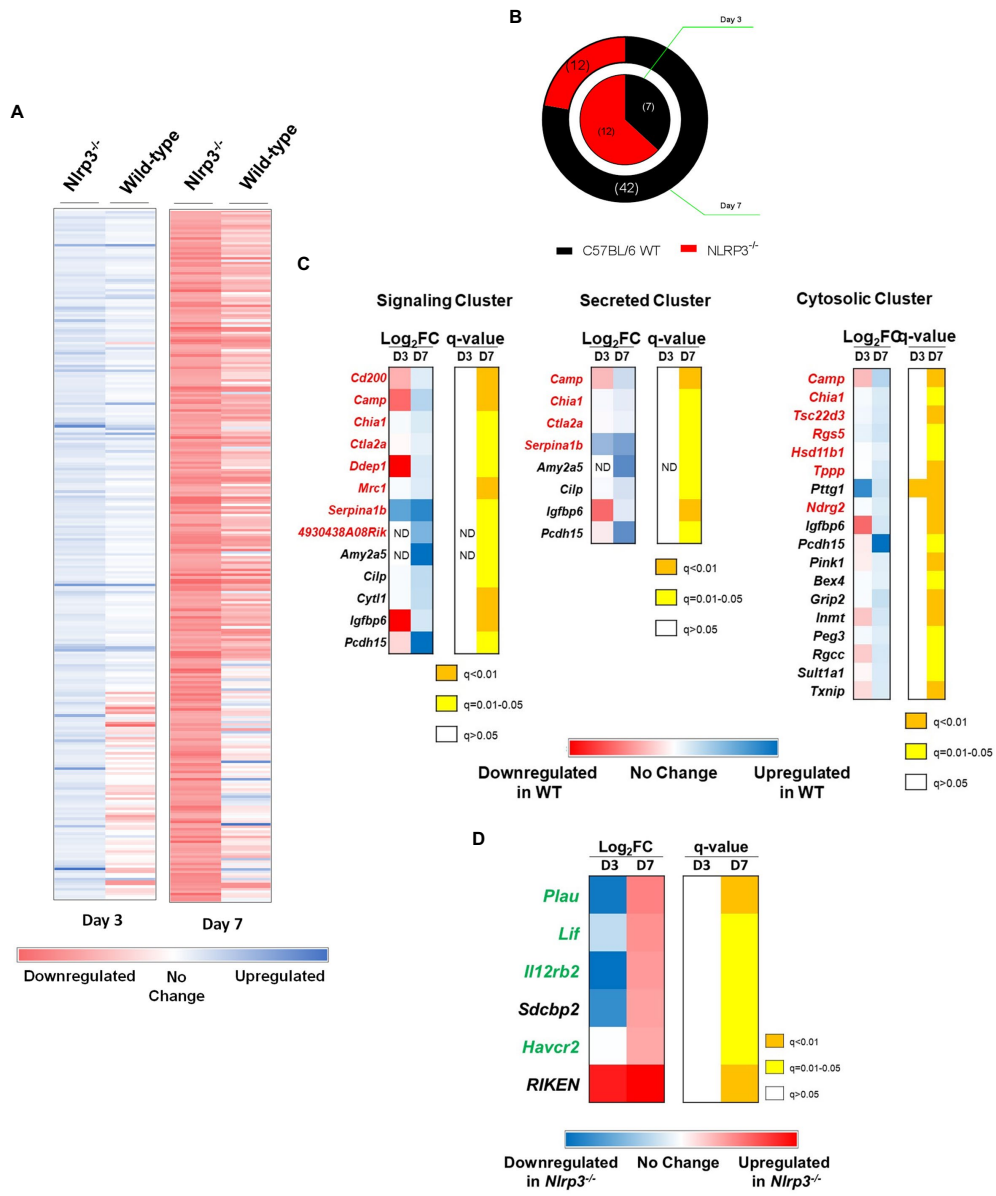
Genes known to enhance the host’s susceptibility to *F. tularensis* infection are downregulated in *Nlrp3^−/−^* mice. Wild-type and *Nlrp3*^−/−^ mice were infected intranasally with 1×10^4^CFU of *F. tularensis* LVS. Total RNA was isolated from lungs of infected mice on days 3 and 7 post-infection and sequenced to determine the expression of various genes in the mouse by RNA-sequencing. The genes were aligned and their expression profile over time has been represented as a heat map of the Log_2_Fold change. **(A)** Expression profile of genes that were significantly differentially expressed (*q*<0.05) in wild-type and *Nlrp3^−/−^* mice. **(B)** Pie chart representing significantly expressed genes between wild-type and *Nlrp3*^−/−^ mice. The inner ring represents the expression profile of the indicated mouse strain on day 3, while the outer ring represents the expression profile on day 7 post-infection. The numbers of differentially expressed genes are shown in the parenthesis. **(C)** DAVID genomics and clusters based on functional annotation of genes in *F. tularensis* LVS-infected wild-type and *Nlrp3^−/−^* mice on day 3 and 7 post-infection. **(D)** Differential expression of genes in *Nlrp3^−/−^* mice known to have a protective role against *F. tularensis* infection. The expression profile is presented as heat maps alongside corresponding *q* values. Genes in red font are known to enhance the susceptibility of the host to *F. tularensis*, while those in green have a protective role. Data shown are cumulative of transcript levels using RNA isolated from lungs of *F. tularensis*-infected mice (*n*=3/time point/group) on days 3 and 7 post-infection.

A total of 10,512 gene transcripts were detected in wild-type and *Nlrp3*^−/−^ mice. A total of 1,426 transcripts were differentially expressed between wild-type and *Nlrp3^−/−^* mice when analyzed based on two or more Log_2_ Fold-change on days 3 and 7 post-infection (Figure 8A). Salmon analysis followed by Wald Test to establish the statistical significance (*q*<0.05) revealed that seven genes were upregulated in wild-type mice on day 3 post-infection, compared to *Nlrp3*^−/−^ mice. On the other hand, 12 genes were significantly upregulated in the *Nlrp3*^−/−^ mice compared to wild-type mice. On day 7 post-infection, 42 genes were significantly upregulated in the wild-type mice, while 12 genes were found to be upregulated in the *Nlrp3*^−/−^ mice (Figure 8B). Of the 19 differentially expressed genes on day 3, only five remained significantly differentially expressed on day 7 post-infection. These included *Nlrp3* and *Pttg1* in wild-type mice and three pseudogenes in *Nlrp3*^−/−^ mice.

We further analyzed the 42 genes that were significantly upregulated in the wild-type mice on day 7 post-infection using DAVID for functional clustering. The software correctly identified 39 of the 42 genes belonging to *Mus musculus*, while three genes were identified as pseudogenes. We constructed a functional annotation chart to ascribe broad functional categories to these 39 genes. The analysis was performed using a threshold count of 2 and 0.1 EASE to determine statistical significance. Thirty-three genes clustered into 19 functional annotation clusters, while six genes (*Gypa*, *Mcmdc2*, *Gm4956*, *Gm15772*, *Smim4*, and *Slc25a34*) could not be assigned to any functional cluster at the set level of stringency.

Based on UniProt Keywords, 13 genes (33.3%) fell into the cluster of signaling-related genes. Of these, 7 (54%) play a deleterious role in response to *Francisella* infection either by conferring anti-inflammatory phenotypes, preventing immune cell activation, or facilitating a silent entry of bacteria (Figure 8C). Nine genes clustered into the functional category of secreted proteins, and of these, four have been reported to have a deleterious role following infection with *Francisella* (Figure 8C). Based on the “Gene Ontology-cellular compartment-direct” (GO_CC_DIRECT) analysis, 19 genes fell into the cytosolic functional cluster, constituting 48.7% of the input genes. Among these, seven genes have detrimental outcomes to *Francisella* infection (Figure 8C). Cumulatively, these results indicate that Nlrp3 alone or in conjunction with unknown protein(s) affect the transcription of various genes in mice infected with *F. tularensis* LVS, which are known to enhance the host’s susceptibility.

### Genes Involved in the Enhancement of Innate Immune Responses Against *F. tularensis* Are Upregulated in *Nlrp3*^−/−^ Mice

Next, we analyzed the significantly upregulated genes in *Nlrp3*^−/−^ mice compared to wild-type mice. Twelve genes were found to be significantly upregulated in the *Nlrp3*^−/−^ mice on day 7 post-infection. When analyzed by DAVID, six of these were pseudogenes, and the DAVID database could not assign those to any functional clusters. The remaining six genes grouped into 12 functional clusters by a threshold count of 2 and an EASE of 0.1 (same as used for wild type mice). The remaining six genes were grouped together in the signaling cluster. Of these, four appear to have a beneficial role during *F. tularensis* infection, either by enhancing the innate immune signaling or by engaging in tissue remodeling to limit tissue damage in the lungs. These genes are implicated in positively affecting the JAK-STAT signaling and regulating IFN-γ production (Figure 8D). Thus, analysis of genes with increased transcript levels in *Nlrp3*^−/−^ mice reveals an increase in host-protective innate immune responses against *F. tularensis*. These results further establish the notion that Nlrp3 dampens the generation of a protective immune response during *F. tularensis* LVS infection.

### The Key Innate Immune Pathway Genes Are Differentially Expressed in *Nlrp3^−/−^* Mice Infected With *F. tularensis* LVS

**Figure 9:**
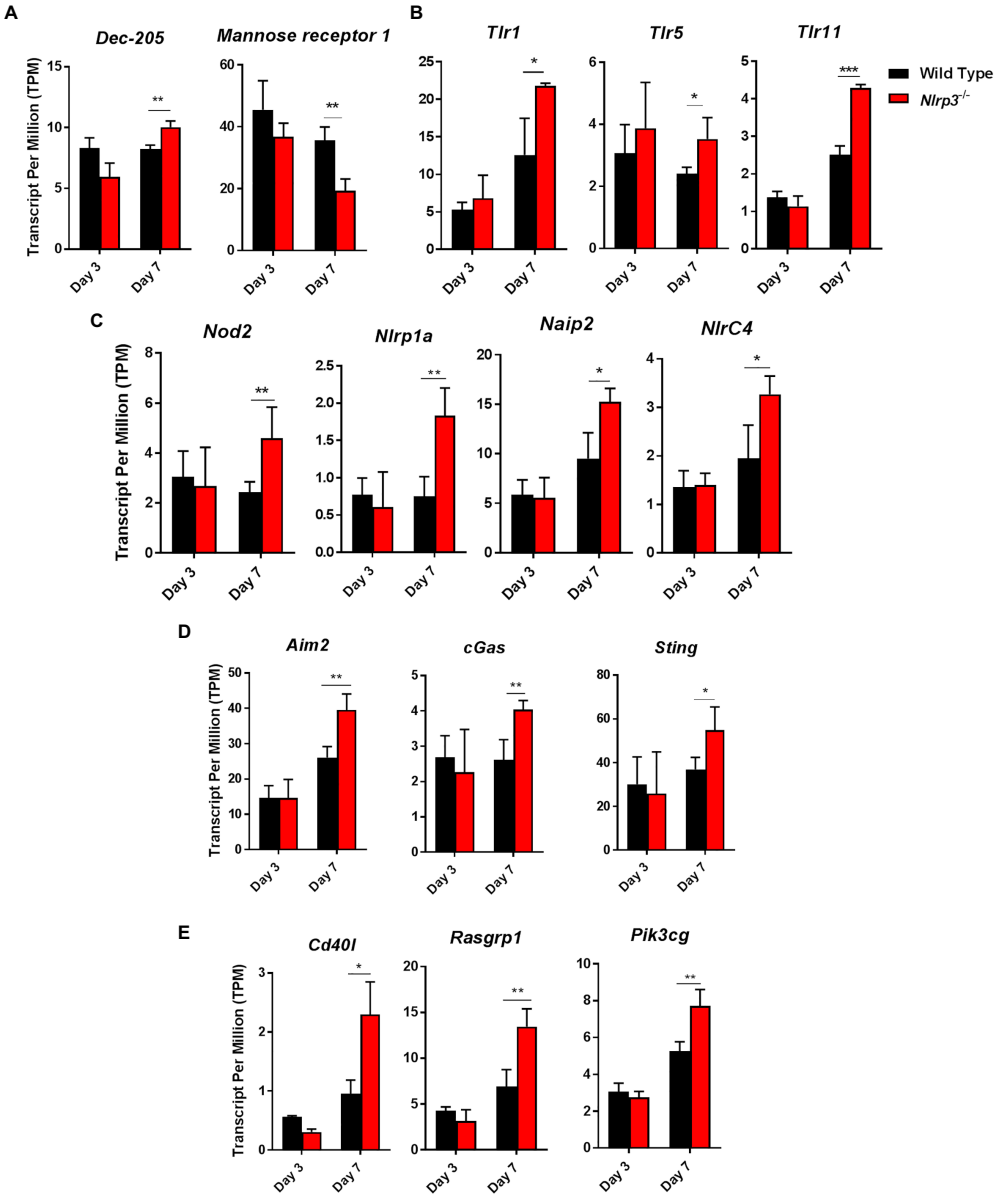
The key innate immune pathway genes are differentially expressed in *Nlrp3^−/−^* infected with *F. tularensis* LVS. Expression levels of C-type lectin receptors (Clrs) **(A)**, *Tlrs* **(B)**, *Nlrs* **(C)**, DNA sensors **(D)**, and components of NF-κβ and MAPK-signaling pathways **(E)** in wild-type and *Nlrp3^−/−^* mice infected with 1×10^4^ CFUs of *F. tularensis* LVS on days 3 and 7 post-infection. Data shown are cumulative of transcript levels using RNA isolated from lungs of *F. tularensis*-infected mice (*n*=3/time point/group) on days 3 and 7 post-infection. The data are presented as transcripts per million (TPM) and analyzed using unpaired *t*-test and a *p*-value of 0.05 or less was considered significant. ^*^p>0.05; ^**^p>0.01; ^***^p>0.001.

Using the RNASeq data, we next determined the expression levels of C-type lectin receptors (Clrs), toll-like receptors (Tlrs), Nlrs, DNA sensors, and components of NF-κβ and MAPK-signaling pathways in wild-type and *Nlrp3^−/−^* mice infected with 1×10^4^ CFUs of *F. tularensis* LVS on days 3 and 7 post-infection. The transcript levels of Clr, *DEC-205* were significantly higher in the *Nlrp3^−/−^* compared to the wild-type mice on day 7 post-infection. However, the reverse was true for mannose receptor 1, with significantly elevated transcript levels were observed in the wild type than *Nlrp3^−/−^* mice on day 7 post-infection (Figure 9A). The transcripts of *Tlr1*, *Tlr5*, *Tlr11*, and *Nod2*, *Nlrp1a*, *Naip2*, and *Nlrc4* were significantly higher in the *Nlrp3^−/−^* than wild-type mice on day 7 post-infection (Figures 9B,C). The transcript levels of DNA sensors *Aim2*, *cGas*, and *Sting* were also significantly elevated in the *Nlrp3*^−/−^ mice on day 7 post-infection than those observed for *F. tularensis* LVS-infected wild-type mice (Figure 9D). A similarly significantly elevated transcript levels were also observed for *CD40L*, *Rasgrp1*, *PI3cKG*, the components of NF-κβ and MAPK-signaling pathways, respectively (Figure 9E). Collectively, these results indicate that the loss of *Nlrp3* is associated with significant upregulation of several innate immune components following *F. tularensis* infection, which in turn allows the generation of a strong protective immune response in the *Nlrp3^−/−^* mice than those observed for the wild-type mice.

## Discussion

The NLR family proteins are important for surveillance of the cytosolic compartment and serve as PRRs for cellular damage as well as infectious agents. The NLRs, NOD1, and NOD2 induce the production of pro-inflammatory cytokines by activating MAPK and NF-κB signaling (Kanneganti, 2010), while the NLRP1, NLRP3, and NLRC4 activate inflammatory caspases by assembling multiprotein inflammasome complex. In addition to these NLRs, a HIN-200 family protein Aim2 results in the assembly and activation of Caspase1 (Fernandes-Alnemri et al., 2010). Aim2, in particular, is important for innate immunity against infection with *F. novicida* (Weiss et al., 2007; Fernandes-Alnemri et al., 2010; Ulland et al., 2010; Peng et al., 2011). However, the contribution of Nlrp3 in innate immune responses against *F. tularensis* remains understudied. Nlrp3 can sense *F. tularensis* LVS and *F. novicida* in human macrophages and leads to assembly and activation of caspase1 with the resultant production of IL-1β (Atianand et al., 2011). The production of IL-18 depends entirely on Aim2 in mice infected with *F. novicida*. The IL-1β response against *F. tularensis* SchuS4 strictly requires Nlrp3, but the requirement for Aim2 is only partial. *Francisella tularensis* LVS requires both Aim2 and Nlrp3 for IL-1β response. However, IL-18 response requires both Aim2 and Nlrp3 following *F. tularensis* LVS as well as *F. tularensis* SchuS4 infections (Atianand et al., 2011; Periasamy et al., 2016). The reasons for selective utilization of Aim2 or Nlrp3 by different *Francisella* strains remain unknown. The objective of this study was to investigate how Nlrp3 contributes to the host’s susceptibility to *F. tularensis* infection.

The results demonstrate that loss of the Nlrp3 is associated with significantly higher levels of bioactive caspase1 and IL-1β. Further, the elevated levels of bioactive IL-1β are also associated with elevated levels of IL-1β and TNF-α, indicating that Nlrp3 dampens macrophage inflammatory response following *F. tularensis* infection. Findings that Nlrp3 deficiency results in significantly higher levels of phospho-IκB, phospho-ERK1/2, and translocation of p65 subunit of NF-κB following infection with *F. tularensis* LVS substantiated the notion that Nlrp3 dampens macrophage response by regulating the NF-κB and MAPK signaling pathways. The *Nlrp3^−/−^* macrophages were better at controlling the growth of *F. tularensis* LVS than the wild type macrophages. Moreover, this feature was specific to *Francisella* and did not extend to *S*. Typhimurium, an unrelated intracellular bacterial pathogen as the *Nlrp3^−/−^* macrophages supported the growth of *S*. Typhimurium similar to the wild-type macrophages. Furthermore, the ability to clear *Francisella* was specific to *Nlrp3^−/−^* macrophages as *F. tularensis* LVS replicated at a higher rate in *Asc^−/−^* and *Caspase-1^−/−^* than the wild-type macrophages. In agreement with the *in vitro* results, the *Nlrp3^−/−^* mice were better at clearing bacteria from the lungs, liver, and spleen. The lower bacterial numbers were also reflected in less severe histopathological lesions. Furthermore, the *Nlrp3^−/−^* mice were less susceptible to a lethal challenge with *F. tularensis* LVS. Similar findings have been reported for NLRP6 and NLRP12 to play a pathogenic role during infection with *Brucella abortus*, *S*. Typhimurium, and *Plasmodium chaubadi* (Anand et al., 2012; Zaki et al., 2014). Collectively, these results demonstrated that Nlrp3 interferes with the clearance of *F. tularensis* by altering the innate immune environment of the infected macrophages and mice.

For clearance of *F. tularensis*, an early pro-inflammatory cytokine response that gears the adaptive immune response toward a Th1 pathway is needed (Chiavolini et al., 2008; Mahawar et al., 2013). *Nlrp3*^−/−^ mice infected with *F. tularensis* LVS had significantly higher levels of IL-1β, IL-6, IL-12p40, G-CSF, GM-CSF, TNF-α, and RANTES as compared to the wild-type mice on day 3 post-infection. These pro-inflammatory cytokines play a crucial role in defense against *F. tularensis* (Roberts et al., 2014). Significantly higher levels of IL-2, IFN-γ, and RANTES were observed in *Nlrp3*^−/−^ mice on day 7 post-infection. These cytokines are required for the development of the adaptive immune response against *F. tularensis*. Moreover, elevated levels of chemokines MIP-1β, KC, and RANTES observed in the lungs of *Nlrp3*^−/−^ mice are responsible for attracting T-cells, dendritic cells, NK cells, and neutrophils to sites of infection. *Nlrp3*^−/−^ mice also had significantly higher anti-inflammatory cytokine IL-10 in the lungs, especially on day 3 post-infection. The cytokine profile observed in mice suggests that Nlrp3 prevents the development of an innate immune environment in the lungs essential for the rapid clearance of *Francisella* and, therefore, enhances the host’s susceptibility to *F. tularensis* infection. An earlier study has reported that Nlrp3 prevents/delays recruitment of mature neutrophils to the lungs of *F. tularensis*-infected mice (Periasamy et al., 2016). Our results further this observation and demonstrate that Nlrp3 prevents the recruitment of mature neutrophils by dampening the pro-inflammatory cytokine response during *Francisella* infection.

The results from this study ruled out that the inflammasome-mediated function of Nlrp3 is not responsible for its detrimental role in tularemia pathogenesis. This prompted us to explore the inflammasome-independent role of Nlrp3 in response to *Francisella* infection. The detrimental role of an exaggerated TH2-biased response in the context of *F. tularensis* infection has been well documented (Kirimanjeswara et al., 2007; Woolard et al., 2007). Previous observations that Nlrp3 drives Th2 polarization by regulating transcription (Bruchard et al., 2015; Ting and Harton, 2015) led us to investigate differences in gene expression profiles between wild-type and *Nlrp3*^−/−^ mice in response to *F. tularensis* LVS infection. Clustering of differentially expressed genes between *F. tularensis*-infected wild-type and *Nlrp3*^−/−^ mice grouped them in the signaling, secreted, and cytosolic categories. Among the signaling cluster, significantly enhanced expression of *Cd200*, *Camp*, *Chia1*, *Ctla2a*, *Dpep1*, *Mrc1*, and *Serpina1b* was observed in the wild-type mice on day 7 post-infection. Specifically, the *CD200* gene product dampens the activation of macrophages and promotes a TH2-biased immune response (Snelgrove et al., 2008; Goulding et al., 2011; Ting and Harton, 2015), which is further amplified by *Chia1* and Dpep1 gene products (Zhu, 2004; Chung et al., 2014). The *Ctla2a* gene product causes T cells to mature into regulatory T cells and thus favors the replication of *F. tularensis* (Sugita et al., 2009; Periasamy et al., 2011). *Mrc1* encodes the mannose receptor, which facilitates silent entry of *F. tularensis*. *Serpina1b* gene products break down neutrophil elastase, while *Camp* encode cathelicidin (Zhang et al., 2005; Schulert, 2006; Barel et al., 2008; Amer et al., 2010). Cathelicidin is bactericidal for *F. novicida*. However, its role against *F. tularensis* is not known (Vonkavaara et al., 2013; Kaushal et al., 2016). *Camp*, *Chia1*, *Ctla2a*, and *Serpina1b* were also clustered in secreted proteins category, indicating these gene products exert their effects within the cells as well as on the bystander cells. In the cytosolic genes cluster, in addition to *Camp* and *Chia1*, *Tsc22d3*, *Rgs5*, *Hsd11b1*, *Tppp*, and *Ndrg2* gene products are all potentially deleterious to the host. *Tsc22d3*, *Rgs5*, *Hsd11b1*, and *Ndrg2* genes have anti-inflammatory effects, whereas *Rgs5* and *Ndrg2* inhibit NF-κB, p38, MAPK, and STAT signaling, which are all critical for effective clearance of *F. tularensis* (Park et al., 2007; Kim et al., 2009; Guo et al., 2013; Wang et al., 2016b). Furthermore, the *Tsc22d3* and *Hsd11b1* genes are negative mediators of pro-inflammatory cytokines IL-1β, IL-6, TNF-α, and IFN-γ (Elenkov, 2004). The *Hsd11b1* gene product leads to the generation of cortisol, which is deleterious to the host during *F. tularensis* infections. Furthermore, *Tppp* promotes tubulin polymerization, which is known to promote *F. tularensis* entry and uptake (Craven et al., 2008b). Having identified genes with deleterious effects, which were all downregulated in the *Nlrp3*^−/−^ mice, we also looked at genes that were upregulated in the *Nlrp3*^−/−^ mice. Overall, very few genes were significantly upregulated and intriguingly, all these genes predicted by DAVID positively regulate JAK-STAT signaling and IFN-γ production. Of these, *Plau*, *Lif*, *Il12rb2*, and *Havcr2* are pro-inflammatory, increase STAT3 signaling, increase the production of acute-phase proteins production and enhance TH1 over TH2 responses. These genes have been implicated in protection against *Mycobacterium tuberculosis* and *Listeria monocytogenes* (Schooltink et al., 1992; Qiu et al., 2012; Quinton et al., 2012; Gorman et al., 2014). Thus, in the presence of Nlrp3, the expression profile is predominated by genes promoting anti-inflammatory TH2 type of immune response. The gene expression profile in the *Nlrp3*^−/−^ mice indicates the development of a strong pro-inflammatory TH1-type response. Collectively, the RNA-seq results suggest that Nlrp3 drastically reduces TH1 immune responses and promotes TH2 responses in *F. tularensis*-infected mice, which impacts bacterial clearance and their survival.

The gene expression profile of the *Nlrp3^−/−^* mice also showed upregulated expression of genes of the innate immune system. The notable ones were surface receptors *Dec-205, Tlr1, Tlr5, Tlr11*, the cytosolic receptors *Nod2, Nlrp1a, Naip2*, the DNA-sensing pathway components *cGas, Sting, Aim2*, and the components of NF-κβ and MAPK-signaling pathway genes *CD40l, Rsagrp1* and *pik3cg*. The transcript levels of all these genes were upregulated in the *Nlrp3^−/−^* mice on day 7 post-infection. Significantly lower levels of Mannose-1 receptor required for the entry of *Francisella* into the cells in *Nlrp3^−/−^* mice indicate that Nlrp3 allows the silent bacterial entry into the cells that may also enhance the host’s susceptibility to infection. This differential regulation of genes could be due to a compensatory mechanism occurring in the *Nlrp3^−/−^* mice as a result of the loss of Nlrp3 or may be due to the role of Nlrp3 as a transcriptional regulator. Evidence is emerging in favor of an immunomodulatory role for Nlrp3. In an ocular infection model of Herpes-Simplex Virus-1, it has been shown that loss of Nlrp3 is associated with increased levels of pro-inflammatory cytokines including IL-1β and TNF-α, increased infiltration of neutrophils, and enhanced Th1 and Th17 cell responses (Gimenez et al., 2016). Although an enhanced inflammatory response in *Nlrp3^−/−^* mice contributes to the severity of the ocular lesions caused by HSV-1 infection, these findings point to the fact that *Nlrp3* plays a prominent role in dampening pro-inflammatory responses. Furthermore, in an experimental allergic airway inflammation model, it has been reported that *Nlrp3^−/−^* mice fail to control eosinophilic infiltration of airways as well as Th2 cytokine and chemokines demonstrating a role of Nlrp3 in the regulation of Th2 responses during allergic lung inflammation (Madouri et al., 2015). It has been proposed that cytoplasmic localization of Nlrp3 promotes inflammasome assembly while its nuclear localization is an indicator of its role as a transcriptional regulator, specifically in Th2 polarization of CD4^+^ T cells. These immunomodulatory roles of Nlrp3 are in concurrence with similar roles described for Nlrp6 and Nlrp12 in response to intracellular bacterial infections (Anand et al., 2012; Lupfer and Kanneganti, 2013; Zaki et al., 2014). As proposed previously, the NLRs can be grouped into two classes based on their functionality. One class consisting of NLRs, such as NOD1, NOD2, and NLRC4, acts by serving as cytosolic sensors that activate pro-inflammatory signaling and clear bacterial pathogens. On the other hand, the other group of NLRs consisting of Nlrp6 and Nlrp12 by serving as molecular switches that silence the TLR-induced inflammatory responses induced consequent to the recognition of bacterial ligands (Lupfer and Kanneganti, 2013). The results from the present study suggest the inclusion of Nlrp3 to the latter group of NLRs that primarily serve to dampen the inflammatory response to minimize tissue damage during *F. tularensis* infection. However, in the process of doing so, it increases the host’s susceptibility to *Francisella*.

To conclude, the present study reports that Nlrp3 suppresses pro-inflammatory responses during *Francisella* infection. The loss of Nlrp3 is associated with enhanced activation of NF-κB and ERK signaling, elevated levels of IL-1β and TNF-α in *F. tularensis*-infected macrophages, and several pro-inflammatory cytokines and chemokines in mice. Moreover, the *Nlrp3^−/−^* macrophages and mice clear *Francisella* more effectively than their corresponding wild-type counterparts and *Nlrp3^−/−^* mice are less susceptible to respiratory tularemia following intranasal infection. This study advances our understanding of the detrimental role of Nlrp3 and demonstrates that Nlrp3 could be a potential target for the development of effective therapeutics for the treatment of tularemia.

## Data Availability Statement

The datasets presented in this study can be found in online repositories. The names of the repository/repositories and accession number(s) can be found at: https://www.ncbi.nlm.nih.gov/, GSE183003.

## Ethics Statement

The animal study was reviewed and approved by Institutional Animal Care and Use Committee (IACUC) of New York Medical College, Valhalla, New York.

## Author Contributions

RS, EB, MH, VR, MA, and WH: experimental performing and analysis. CB and MM: experimental design and funding. RS, CB, and MM: manuscript preparation. All authors contributed to the article and approved the submitted version.

## Funding

This work was supported, in whole or part, by National Institutes of Health Grants R56AI101109, R21AI51277 (CB), and R15AI107698 (MM).

## Conflict of Interest

The authors declare that the research was conducted in the absence of any commercial or financial relationships that could be construed as a potential conflict of interest.

## Publisher’s Note

All claims expressed in this article are solely those of the authors and do not necessarily represent those of their affiliated organizations, or those of the publisher, the editors and the reviewers. Any product that may be evaluated in this article, or claim that may be made by its manufacturer, is not guaranteed or endorsed by the publisher.
