# Supplementary material for: Nlrp3 Increases the Host’s Susceptibility to Tularemia
Source: Front Microbiol. 2021 Oct 6;12:725572. doi: 10.3389/fmicb.2021.725572 (PMC8527020; doi:10.3389/fmicb.2021.725572)
Supplement: Supplementary file 1 [file Data_Sheet_1.PDF]

Figure S1

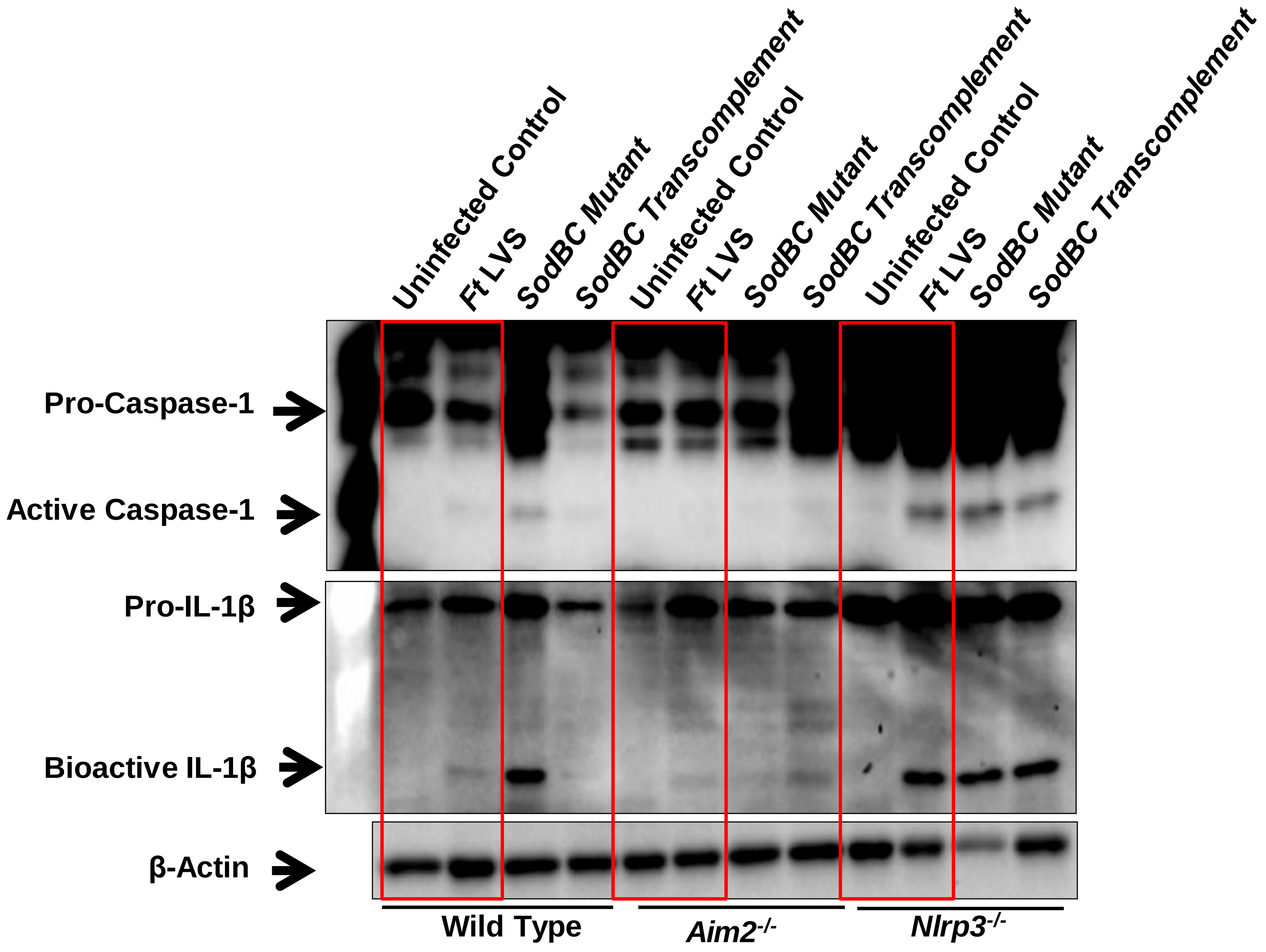

Figure S1 Nlrp3 dampens pro-inflammatory cytokine response. Wild type, *Aim2*$^{-/-}$ and *Nlrp3*$^{-/-}$ macrophages were infected with wild type *F. tularensis* LVS, the *sodBC* mutant and the *sodBC* transcomplemented strain of *F. tularensis* LVS at an MOI of 100. The cell lysates were prepared 24 hours post-infection and western blot analysis was performed to determine levels of bioactive caspase1 and IL-1β. The blot was stripped and re-probed with anti-β-actin antibodies to determine the equal loading of all the samples. The *sodBC* mutant deficient in the antioxidant enzymes superoxide dismutase B and C and its transcomplemented strain were used as a controls (Melillo et al., 2010). The western blot images are representative of three independent experiments. The red boxes indicate the cropped version of this image shown in Figure 1A.

Melillo, A. A., Bakshi, C. S., & Melendez, J. A. (2010). Francisella tularensis antioxidants harness reactive oxygen species to restrict macrophage signaling and cytokine production. *Journal of Biological Chemistry*, *285*(36), 27553–27560. https://doi.org/10.1074/jbc.M110.144394
